# Bioactive Sulfated Polysaccharides from Green Algae *Codium tomentosum* Stackhouse, 1797: Structural Characterization and Therapeutic Potential

**DOI:** 10.3390/ijms27114848

**Published:** 2026-05-27

**Authors:** Bouchra Benhniya, Fatima Zahra Karmil, Soukaina El Maliki, Noureddine El Hasbaoui, Fatima Lakhdar, Christel Marty, David Boutolleau, Nathalie Bourgougnon, Samira Etahiri

**Affiliations:** 1 Laboratory of Marine Biotechnologies and Environment, Faculty of Sciences, Chouaib Doukkali University, BP 20, El Jadida 24000, Morocco; 2 Laboratory of Physical Chemistry of Materials, Department of Chemistry, Chouaib Doukkali University, BP 20, El Jadida 24000, Morocco; 3 Coordination and Analytical Chemistry Laboratory (LCCA), Department of Chemistry, Faculty of Sciences, Chouaib Doukkali University, BP 20, El Jadida 24000, Morocco; 4 EMR CNRS, LBCM, IUEM, Université Bretagne Sud, 56000 Vannes, France; 5 INSERM, Virology Department, Sorbonne Public Health Institute, AP-HP, Hôpitaux Universitaires Pitié—Charles Foix, National Reference Centre for Herpesviruses, Sorbonne Université, 75013 Paris, France

**Keywords:** *Codium tomentosum*, sulfated polysaccharides, antiviral activity, HSV1, antioxidant activity, antimicrobial activity, marine seaweed, bioactive compounds

## Abstract

Marine seaweeds are recognized as a rich source of structurally diverse bioactive compounds, particularly sulfated polysaccharides with promising biomedical applications. In the present study, a sulfated polysaccharide *Codium tomentosum* fraction (PSCT) was extracted from the Moroccan green seaweed *Codium tomentosum* and subjected to comprehensive chemical, structural, and biological characterization. The extraction yield reached 22.01%, and the polysaccharide fraction was mainly composed of neutral sugars (66.84%), along with significant levels of sulfate groups (8.73%) and uronic acids (4.13%). Monosaccharide analysis revealed a predominance of galactose and arabinose, indicating a complex heteropolysaccharide structure. Spectroscopic and morphological analyses (FTIR, XRD, UV–Vis, and SEM–EDS) suggested predominantly amorphous characteristics and confirmed the sulfated profile of the extract. Biological evaluation demonstrated that PSCT exhibits multifunctional bioactivities. The extract showed notable antiviral activity against Herpes Simplex Virus 1 (HSV1), with an EC_50_ value of 12.22 ± 2.90 µg/mL and no detectable cytotoxicity (CC_50_ > 200.00 µg/mL). In addition, PSCT displayed strong antimicrobial activity against *Staphylococcus aureus*, *Escherichia coli*, and *Candida albicans*, with remarkably low MIC values ranging from 0.05 to 0.78 mg/mL. Furthermore, antioxidant assays revealed concentration-dependent activity, with up to 68% DPPH radical scavenging and an IC_50_ of 1.25 mg/mL, indicating a moderate antioxidant potential, along with notable reducing power in the FRAP assay. Overall, these findings highlight the potential of *C. tomentosum*-derived polysaccharides as multifunctional natural agents with antiviral, antimicrobial, and antioxidant properties, supporting their prospective application in pharmaceutical and biomedical fields.

## 1. Introduction

Seaweeds are a diverse group of marine macroalgae widely distributed across aquatic ecosystems worldwide [1,2]. These organisms are continuously exposed to dynamic and often extreme environmental conditions, including fluctuations in salinity, temperature, light intensity, and nutrient availability. As a result, seaweeds have developed complex metabolic and adaptive systems that enable their survival in such environments. These evolutionary adaptations are closely associated with the biosynthesis of a wide variety of structurally complex and biologically active metabolites, positioning seaweeds among the most important natural reservoirs of bioactive compounds with applications in pharmaceutical, nutraceutical, and industrial fields [3,4]. Moreover, prolonged environmental pressures have driven seaweeds to produce unique metabolites rarely found in terrestrial organisms, contributing to their ecological defense mechanisms and increasing their biotechnological value [5].

Among seaweeds, green algae (Chlorophyta) represent an important group characterized by high growth rates, wide geographical distribution, and rich biochemical profiles. Within this group, the genus *Codium* (family *Codiaceae*, order Bryopsidales) has gained particular attention due to its ecological importance and biochemical diversity [6,7]. This genus, commonly known as “dead man’s fingers” because of its spongy and finger-like morphology, includes more than 140 species inhabiting diverse marine environments [8,9]. Importantly, *Codium* species are recognized for their capacity to produce a wide range of bioactive compounds, including pigments, phenolics, vitamins, minerals, and sulfated polysaccharides [10,11].

In particular, *C. tomentosum* Stackhouse 1797, characterized by its velvety texture and bushy morphology, has a thallus composed of cylindrical dichotomously branched siphons. The terminal parts of these siphons develop into swollen utricles, which are observable under the microscope. These utricles are characterized by the presence of a cap-like apical structure called a spine or mucron [12]. This species has been traditionally used as a food additive and animal feed, particularly in Southeast Asia, due to its nutritional value and bioactive composition [13]. Despite these promising characteristics, this species remains relatively underexplored compared to other members of the genus, such as *C. fragile*, *C. decorticatum*, and *C. divaricatum* [14,15], highlighting the need for further investigation into its biological and biomedical potential.

Polysaccharides constitute one of the major components of seaweed biomass and play crucial roles in both structural integrity and energy storage [16,17]. These cell wall macromolecules are composed of diverse monosaccharides, including galactose, fucose, xylose, rhamnose, and uronic acids, and are often highly sulfated, which contributes to their unique physicochemical and biological properties [1,18]. In green algae, sulfated homo- and heteropolysaccharides exhibit significant structural diversity, including variations in glycosidic linkages, branching patterns, and sulfate group distribution [14,19,20]. From a biochemical perspective, *C. tomentosum* is notable for the absence of cellulose in its cell wall and the presence of distinct sulfated polysaccharides such as arabinans, arabinogalactans, and mannans [13,21]. This diversity is directly associated with their wide range of biological activities.

Consequently, numerous studies have reported that seaweed-derived polysaccharides possess a broad spectrum of bioactivities, including anti-inflammatory, antioxidant, antimicrobial, antidiabetic, anticoagulant, and anticancer [22,23,24,25,26,27,28]. More importantly, increasing attention has been directed toward their antimicrobial and antiviral potential. Sulfated polysaccharides have demonstrated the ability to inhibit critical steps of pathogen development, including microbial adhesion, viral entry, fusion, and replication [29,30]. Furthermore, structure–activity relationship studies have shown that their biological efficacy is strongly influenced by parameters such as monosaccharide composition, degree of sulfation, molecular weight, and conformational flexibility [18,31].

In parallel, the global burden of infectious diseases has significantly increased in recent decades, driven by the emergence of new pathogens and the spread of antimicrobial resistance [32,33]. Viral infections, notably those caused by Herpes Simplex Virus 1 (HSV1), remain major public health concerns due to their high prevalence and ability to establish lifelong latency [34]. Although antiviral drugs such as acyclovir are widely used, their effectiveness is increasingly limited by the emergence of resistant strains [35]. Similarly, bacterial and fungal infections are becoming increasingly difficult to treat due to the rapid spread of antimicrobial resistance. This phenomenon is driven by multiple factors, including the misuse of antibiotics, genetic adaptation, and horizontal gene transfer among microorganisms [36,37]. In addition, fungal pathogens have shown increasing resistance to conventional antifungal therapies, particularly in immunocompromised patients [38]. These challenges highlight the urgent need for new antimicrobial agents with improved efficacy and broader mechanisms of action.

Furthermore, infections caused by viruses, bacteria, and fungi are frequently associated with oxidative stress, characterized by the excessive production of reactive oxygen species (ROS) [39]. Elevated ROS levels can damage essential biomolecules, including lipids, DNA, and proteins, leading to cellular dysfunction and the development of various diseases. In the context of HSV1 infection, oxidative stress has been shown to promote viral replication and disrupt cellular processes [40]. In light of these considerations, the present study aims to extract and chemically characterize sulfated polysaccharides derived from *C. tomentosum* and to investigate their biological activities, including in vitro antiviral activity against HSV1, in addition to antimicrobial and antioxidant effects. This work seeks to provide new insights into the pharmacological potential of this green seaweed as a promising source of natural bioactive compounds.

## 2. Results and Discussion

### 2.1. Structural Characterization of PSCT

#### 2.1.1. Extraction Yield, Chemical Analysis, and Monosaccharide Composition

The recovered PSCT fraction with a yield of 22.01 ± 0.03% (*w*/*w*), indicating efficient retrieval of water-soluble biomolecules under the applied conditions (Table 1). Comprehensive chemical analysis confirmed that PSCT is predominantly composed of neutral sugars (66.84 ± 0.03%), consistent with a polysaccharide-rich matrix, while the notable sulfate content (8.73 ± 0.16%) is consistent with sulfated polysaccharides. The moderate uronic acid level (4.13 ± 0.05%) points to the incorporation of acidic sugar residues, while the low protein content (1.14 ± 0.10%) implies minimal contamination by proteinaceous material following the extraction process type [41,42]. The monosaccharide composition revealed a heterogeneous sugar profile dominated by galactose (51.88 ± 0.13%) and arabinose (32.64 ± 2.08%), indicating the prevalence of arabinogalactan-type polysaccharides. This was accompanied by xylose (4.28 ± 0.22%) and rhamnose (2.31 ± 0.64%) as minor neutral sugars, together with glucuronic acid (6.62 ± 1.18%) and trace amounts of fructose (0.93 ± 0.03%) (Table 1).

This profile aligns with the known cell wall architecture of *Codium* species, which typically feature arabinogalactan-type polymers or sulfated rhamnan–xylan [43]. The co-occurrence of sulfate esters, uronic acids, and diverse neutral monosaccharides supports the hypothesis that PSCT possesses a complex, branched heteropolysaccharide structure, potentially underpinning the multifunctional bioactivity (antiviral, antimicrobial, antioxidant) observed in subsequent assays [44,45].

#### 2.1.2. XRD, FT-IR, and UV-Vis Analysis

**Figure 1:**
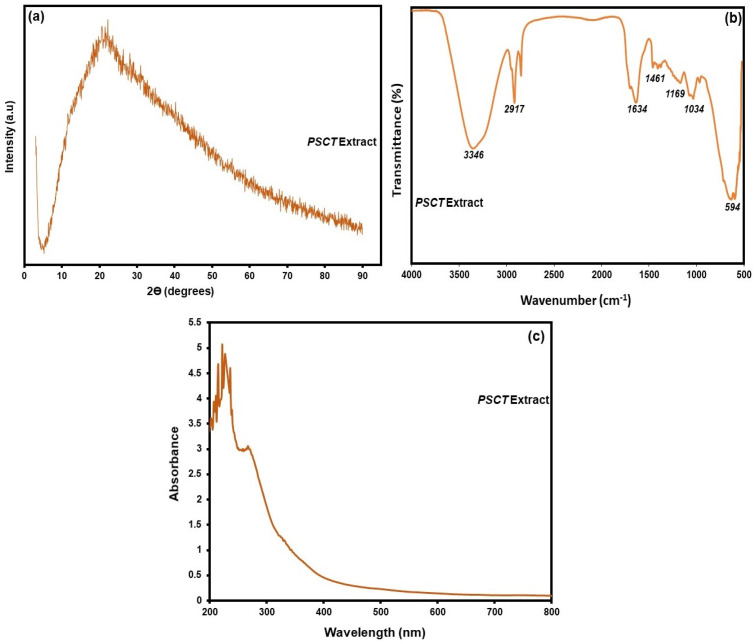
XRD patterns (**a**) of PSCT. FTIR spectra (**b**) of PSCT extract. UV–Vis absorption spectrum (**c**) of PSCT.

The X-ray diffraction pattern of PSCT extract exhibited a characteristic broad diffraction profile with a prominent amorphous halo centered at approximately 19.7° (2θ), accompanied by a less intense shoulder around 22.5° (2θ) (Figure 1a). The complete absence of sharp, well-defined crystalline peaks across the scanned 2θ range (5–80°) suggests predominantly amorphous characteristics of the extracted material. This diffuse scattering pattern is indicative of limited long-range molecular ordering within the polysaccharide matrix, a structural feature commonly observed in sulfated glycans derived from marine green algae [11,46]. The broad maximum near 20° corresponds to the characteristic intermolecular spacing of hydrated (Figure 1a) polysaccharide chains arranged in a disordered manner, reflecting the heterogeneous composition revealed through monosaccharide analysis.

The semi-crystalline character suggested by the subtle shoulder at 22.5° may arise from localized regions where xylose and rhamnose units adopt transient ordered arrangements, although these domains remain insufficiently organized to generate distinct Bragg reflections (Figure 1a). This structural disorder likely stems from the irregular distribution of sulfate ester groups (8.73%) along the polysaccharide backbone, which disrupts chain packing and prevents extensive crystallization [11,46]. The amorphous architecture observed in PSCT aligns with XRD patterns reported for sulfated polysaccharides from other *Codium* species, where high sulfate content and diverse monosaccharide composition inherently limit crystalline domain formation [9]. From a functional perspective, this non-crystalline structure may enhance water solubility and increase the accessibility of bioactive functional groups, potentially facilitating interactions with viral particles or microbial cell walls.

The FTIR spectrum of PSCT demonstrated characteristic absorption bands consistent with sulfated heteropolysaccharides (Figure 1b). A significant, intense absorption band centered at approximately 3346 cm^−1^ was attributed to O–H stretching vibrations of hydroxyl groups from sugar units, indicating extensive hydrogen bonding within the polysaccharide matrix [47,48]. The weak band near 2917 cm^−1^ corresponded to C–H stretching vibrations of aliphatic CH_2_ groups present in the carbohydrate backbone. The absorption peak at 1634 cm^−1^ was assigned to bound water molecules and carboxylate groups (C=O stretching) from uronic acids, while the band at 1461 cm^−1^ reflected C–H bending vibrations [49]. Critically, the absorption bands at 1169 cm^−1^ were characteristic of S=O and C–O–S stretching vibrations, confirming the presence of sulfate ester groups (8.73%) identified in the chemical analysis. The strong absorption around 1052 cm^−1^ corresponded to C–O–C glycosidic linkage vibrations, indicative of the polysaccharide structure. Additional bands in the fingerprint region (1034–594 cm^−1^) suggested the presence of rhamnose and xylose units, consistent with the monosaccharide composition (Table 1). The overall spectral profile confirmed the sulfated polysaccharide nature of PSCT and supported the structural complexity inferred from chemical characterization [50]. However, some limitations should be acknowledged. Molecular weight determination and molecular weight distribution analyses were not performed in the present study due to technical limitations, although these parameters are known to strongly influence polysaccharide bioactivity. In addition, while FTIR analysis confirmed the presence of sulfate ester groups, the precise sulfate substitution positions could not be identified because advanced structural analyses such as NMR or desulfation–methylation were beyond the scope of this work. Therefore, further investigations involving detailed structural characterization will be necessary to better understand the structure–activity relationships of PSCT.

The UV-Visible absorption spectrum of PSCT extract revealed strong absorption in the ultraviolet region with maximum peaks around 205–210 nm and 260–280 nm, followed by a progressive decline in absorbance across the visible range (400–800 nm) (Figure 1c). The intense absorption band near 205 nm was attributed to the transitions of carbonyl groups and peptide bonds within the polysaccharide–protein complex, consistent with the carbohydrate backbone structure identified in the chemical analysis [11]. The absorption maxima observed between 260 and 280 nm corresponded to electronic transitions of aromatic rings, suggesting the presence of phenolic compounds, nucleic acid contaminants, or aromatic amino acids from residual proteins [49]. The absence of significant absorption beyond 400 nm indicated minimal pigmentation in the aqueous extract, confirming effective removal of chlorophyll and carotenoid pigments during the extraction process [46]. The characteristic UV absorption profile, with prominent bands in the 200–300 nm range, aligns with spectra reported for sulfated polysaccharides from green marine algae and supports the presence of conjugated functional groups that may contribute to the extract’s antioxidant capacity through electron donation mechanisms [11]. The gradual baseline shift toward longer wavelengths reflected the typical light scattering behavior of macromolecular polysaccharide solutions.

#### 2.1.3. SEM-EDS Analysis

**Figure 2:**
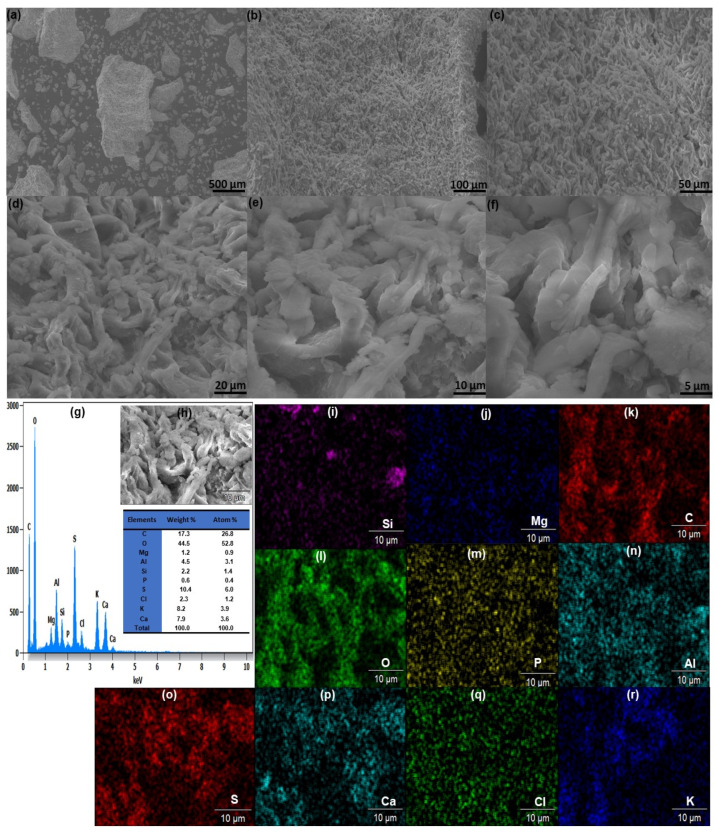
SEM images (**a**–**f**) of the PSCT. EDS spectrum of the PSCT (**g**), morphology (**h**), and (**i**–**r**) elemental maps.

SEM analysis revealed distinct morphological features of the *C. tomentosum* polysaccharide fraction. At lower magnifications, the extract appeared as irregular, densely packed aggregates, while higher-resolution imaging (5–20 µm) exposed a highly porous, fibrous architecture composed of interwoven, sheet-like lamellae (Figure 2a–f). This amorphous, non-uniform surface topology aligns with the broad diffraction halo observed in XRD analysis and suggests a loosely organized macromolecular network with substantial free volume [12]. Energy-dispersive X-ray spectroscopy confirmed the elemental profile, with oxygen (44.5 wt%) and carbon (17.3 wt%) dominating the spectrum, consistent with the carbohydrate-rich composition determined through chemical analysis (Figure 2g,h). A pronounced sulfur signal (10.4 wt%, 6.0 at%) was detected, directly corroborating the sulfate content (8.73%) and confirming the integration of esterified sulfate groups within the polysaccharide matrix. Additional elements, including potassium (8.2 wt%), calcium (7.9 wt%), chloride (2.3 wt%), magnesium (1.2 wt%), and phosphorus (0.6 wt%) were identified, reflecting the natural ionic composition of marine green algae and residual mineral phases.

Elemental mapping (Figure 2i–r) demonstrated homogeneous distribution of carbon, oxygen, and sulfur across the analyzed region, indicating uniform sulfation throughout the polymer framework. Potassium and calcium exhibited widespread dispersion, likely participating in electrostatic interactions with uronic acid carboxylate and hydroxyl moieties. Aluminum and silicon showed localized accumulation patterns, which may correspond to trace silicate minerals inherent to the algal cell wall or minor contributions from sample preparation substrates [12]. The combined morphological and elemental data confirm that the aqueous extraction preserved a structurally coherent, sulfated heteropolysaccharide network with characteristic marine mineral associations, providing a physicochemical basis for the extract’s solubility, stability, and subsequent biological interactions.

### 2.2. Biological Activity

#### 2.2.1. Antiviral Activity

The antiviral activity of PSCT was evaluated for its potential to inhibit HSV1 in vitro using Vero cells, as determined based on cell viability (measured over 72 h). At all treated concentrations, no cytotoxicity was detected after the 72 h period, indicating that PSCT is a good source of biologically compatible materials (Table 2). The PSCT extract demonstrated strong inhibition of HSV1 viral replication, with an EC_50_ (Effective Concentration for 50% inhibition) of 12.22 ± 2.9 μg/mL at an MOI (Multiplicity of Infection, corresponding to the ratio between the number of infectious viral particles and the number of target cells) of 0.001 ID_50_/cell.

The antiviral effects observed in this study correspond to many earlier studies that suggested that the polysaccharides derived from marine algae could serve as a therapeutic agent for viral infections. Numerous studies have demonstrated the broad-spectrum antiviral potential of algal polysaccharides against viruses such as human immunodeficiency virus, dengue virus, influenza virus, and measles virus [30,51,52,53,54].

Antiviral activity is strongly influenced by their chemical composition, which plays a key role in modulating their biological properties. The structural characteristics that affect this activity likely include the degree of sulfate substitution, molecular weight, and type of monosaccharide [18,55,56]. At the mechanistic level, sulfated polysaccharides are known to interfere with multiple stages of the viral life cycle. They can inhibit viral attachment and entry by competing with viral glycoproteins for binding to host cell receptors [1,26]. In addition, they may affect intracellular stages, including viral replication, transcription, and protein synthesis.

Sulfated polysaccharides from green algae, including *Monostroma nitidum*, *Caulerpa brachypus*, *C. fragile*, and *Chaetomorpha* species, have shown strong anti-HSV1 inhibitory activity during viral attachment, with IC_50_ values ranging from 0.38 to 8.5 µg/mL. Comparatively, extracts from *Cladophora* species showed variable antiviral effects depending on treatment timing, with EC_50_ values of 70.31 µg/mL (pre-treatment) and 9.78 µg/mL (post-treatment) [57]. Ulvan isolated from the green algae *Ulva pertusa* has been shown to inhibit HSV1 infection by blocking viral adsorption and replication [58]. Likewise, chemically modified ulvan exhibited enhanced antiviral activity when applied post-infection, suggesting inhibition of viral DNA replication and protein synthesis, as confirmed through immunofluorescence and RT-PCR analyses [59].

Sulfated polysaccharides isolated from *Caulerpa racemosa* have demonstrated potent antiviral activity against HSV1, including acyclovir-resistant strains, with EC_50_ values ranging from 2.2 to 4.2 µg/mL [60]. Similarly, polysaccharides from *Caulerpa brachypus* exhibited antiviral activity (EC_50_ = 9.6 µg/mL), with efficacy closely related to the degree of sulfation and monosaccharide composition [60].

In the same context, Ohta et al. [61] reported that a sulfated galactan isolated from *C. fragile*, mainly composed of galactose and containing approximately 11% sulfate groups, exhibited significant antiviral activity against HSV2. This activity was attributed to its ability to interfere with early stages of viral infection, particularly adsorption and penetration into host cells, and was further supported by in vivo results showing reduced viral load and improved clinical outcomes. Higher glucose content, as reported in *C. fragile* extracts, has been associated with enhanced antiviral activity [62]. Moreover, inhibition of HSV1 replication has been linked to interference with carbohydrate metabolism, as demonstrated by the ability of 2-deoxy-D-glucose to reduce viral production by up to 98% [24].

In addition, highly sulfated polysaccharides rich in galactose have been reported to exhibit strong antiviral effects by blocking viral adsorption and entry into host cells [53]. In this context, the antiviral activity of the PSCT extract (EC_50_ = 12.22 µg/mL) may be attributed to the combined effect of its sulfate groups and galactose-rich composition, supporting a clear relationship between structure and antiviral activity. Compared with these studies, PSCT showed comparable antiviral activity, placing it within the range of bioactive polysaccharides derived from green algae. Overall, these results suggest that PSCT from *C. tomentosum* is a promising natural antiviral agent against HSV1. Further studies are required to isolate and characterize the active compounds and to elucidate their precise mechanisms of action at the molecular level.

#### 2.2.2. Antimicrobial Activity

**Figure 3:**
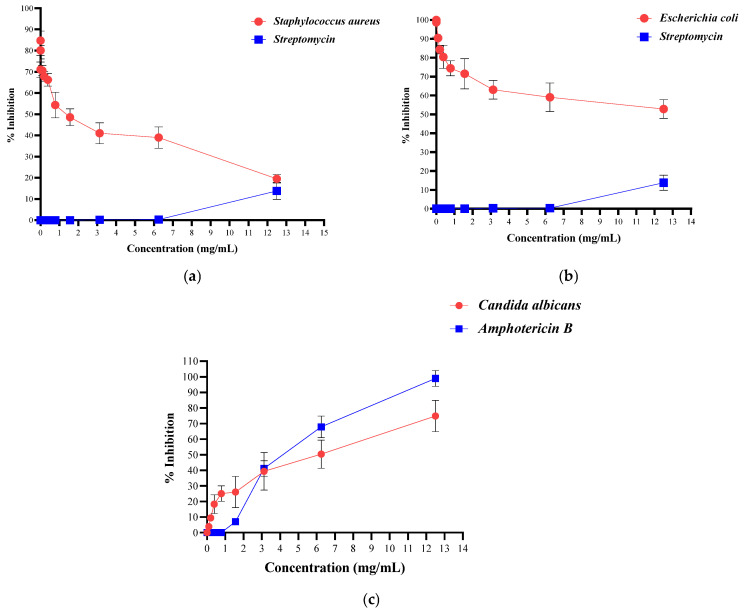
Kinetics of microbial inhibition induced by PSCT. (**a**) *S. aureus*, (**b**) *E. coli*, and (**c**) *C. albicans*.

Sulfated polysaccharides have attracted massive interest in recent years because of their broad spectrum of antimicrobial activity. In the present study, the antimicrobial activity of PSCT extracted from *C. tomentosum* was evaluated against *S. aureus*, *E. coli*, and *C. albicans*. The inhibitory effects of PSCT on microbial growth are presented in Figure 3, showing a concentration-dependent response that varied according to the tested microorganism.

Both *S. aureus* and *E. coli* exhibited pronounced sensitivity to PSCT at low concentrations, with inhibition rates reaching approximately 80–90%. Interestingly, a reduction in inhibitory activity was observed at higher concentrations. This non-linear response may be attributed to molecular aggregation phenomena, reduced bioavailability of active compounds, or adaptive bacterial responses [55,63]. In contrast, streptomycin displayed a conventional dose-dependent increase in antibacterial activity. These findings suggest that PSCT exhibits a strong antimicrobial effect at low concentrations, highlighting its potential as an alternative antimicrobial agent.

For antifungal activity, PSCT demonstrated a clear dose-dependent inhibitory effect against *C. albicans*, with maximal inhibition reaching approximately 70–75%. Although amphotericin B exhibited higher antifungal potency, the observed activity of PSCT remains significant and consistent with previous reports on marine-derived bioactive compounds [64]. The minimum inhibitory concentrations (MICs) of PSCT are summarized in Table 3.

The MIC values obtained (0.05–0.78 mg/mL) indicate strong antimicrobial efficacy. The lowest MIC was recorded against *C. albicans* of 0.05 ± 0.01 mg/mL, suggesting high susceptibility of the yeast. *S. aureus* also exhibited notable sensitivity, whereas *E. coli* showed comparatively lower susceptibility. The reduced sensitivity of *E. coli* can be attributed to the structural complexity of Gram-negative bacteria, particularly the presence of an outer membrane rich in lipopolysaccharides, which limits the penetration of antimicrobial agents [65].

Sulfated polysaccharides, in particular, may inhibit microbial growth via several pathways, such as preventing microbial adhesion, increasing membrane permeability, inducing leakage of intracellular constituents, and inhibiting protein and nucleic acid synthesis. Additionally, their anionic nature may reduce nutrient availability by chelating essential ions in the extracellular environment [66]. In agreement with these mechanisms, previous studies have reported similar antimicrobial activities in marine algae, including *Ulva reticulata* [67] and *Chlamydomonas reinhardtii* [68]. The biological activity of polysaccharides exhibits significant variation dependent on the following structural characteristics: molecular mass, number of sulfate groups attached to the molecule, and types of sugars contained in the molecules [69].

The observed antimicrobial activity may be associated with not only the presence of sulfated polysaccharides but also phenolic compounds and terpenoids commonly identified in *Codium* species. These compounds are known to exert their effects through multiple mechanisms, including disruption of cell membranes, interaction with membrane sterols, and interference with essential cellular processes [70].

Compared with these reported studies, the PSCT extract evaluated in the present work exhibited significantly lower MIC values (0.05–0.78 mg/mL), indicating a markedly higher antimicrobial potency. This enhanced activity may be attributed to differences in chemical composition, degree of sulfation, molecular weight distribution, and structural conformation of the polysaccharide fraction. Overall, the present findings suggest that PSCT from *C. tomentosum* is a promising natural antimicrobial agent with strong activity at low concentrations.

#### 2.2.3. Antioxidant Activity

**Figure 4:**
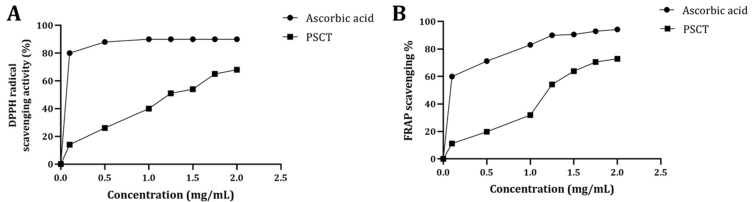
Antioxidant activity of PSCT evaluated using (**A**) the DPPH radical scavenging assay and (**B**) the ferric reducing antioxidant power (FRAP) assay, in comparison with ascorbic acid as a standard antioxidant.

The antioxidant activity of PSCT was evaluated using both DPPH radical scavenging and FRAP assays (Figure 4), revealing a clear concentration-dependent effect in both systems. In the DPPH assay, PSCT exhibited a progressive increase in radical scavenging activity, reaching approximately 68% inhibition at 2 mg/mL, indicating a strong capacity to neutralize free radicals. The calculated IC_50_ value was 1.25 mg/mL, reflecting a moderate but significant antioxidant potential. Similarly, the FRAP assay showed a marked increase in reducing power, with PSCT achieving up to 72.82% at the highest tested concentration. Although ascorbic acid demonstrated higher antioxidant activity across all concentrations, PSCT displayed substantial and consistent activity in both assays, confirming its ability to act as an effective electron donor and reducing agent.

The antioxidant activity of PSCT observed in the present study is in agreement with previous reports on sulfated polysaccharides derived from green algae (*Chlorophyta*), which are widely recognized for their radical scavenging and reducing properties. Several studies have demonstrated that polysaccharides from species within this group exhibit significant antioxidant potential. For instance, polysaccharides extracted from *Caulerpa lentillifera* showed notable DPPH radical scavenging activity, which was closely related to their sulfate and uronic acid content [71]. Similarly, a polysaccharide fraction from *Caulerpa racemosa* exhibited antioxidant activity with an IC_50_ value exceeding 2 mg/mL, highlighting the influence of both sulfate groups and associated polyphenolic compounds [72]. In addition, Magdugo et al. [60] reported that a sulfated polysaccharide isolated from *C. racemosa*, mainly composed of galactans and mannans (15.36–32.71% of dry weight), possessed significant antioxidant and radical scavenging activities.

Comparable activities have also been described for ulvan extracted from *Ulva intestinalis*, which demonstrated strong DPPH scavenging capacity and reducing power [73], as well as for polysaccharides derived from *Ulva rigida*, where enhanced antioxidant activity was associated with extraction efficiency and structural characteristics [74]. Moreover, polysaccharide-rich fractions from *Udotea flabellum* showed selective metal chelation properties, particularly for copper ions, further contributing to their antioxidant behavior [75].

In agreement with these findings, sulfated polysaccharides isolated from *Codium* species have been reported to possess significant antioxidant and radical scavenging activities [26], which reinforces the relevance of the results obtained in the present study for PSCT. The antioxidant mechanisms of algal polysaccharides are multifactorial and primarily related to their chemical structure. Functional groups such as hydroxyl (–OH) and carboxyl (–COOH) groups play a crucial role in electron donation, enabling the neutralization of reactive oxygen species (ROS), including superoxide, hydroxyl, and peroxyl radicals [76]. In addition, these polysaccharides can chelate transition metal ions such as iron and copper, thereby limiting the formation of highly reactive radicals through Fenton-type reactions [77]. This dual action, radical scavenging and metal chelation, contributes significantly to their overall antioxidant capacity.

Beyond direct chemical interactions, polysaccharides may also exert protective effects at the cellular level. They can prevent lipid peroxidation and preserve membrane integrity by stabilizing lipid radicals. Furthermore, they are known to modulate key signaling pathways involved in oxidative stress response, particularly the Nrf2 pathway, which regulates the expression of antioxidant enzymes. For example, polysaccharides from *Enteromorpha prolifera* have been shown to enhance antioxidant defenses via activation of the Nrf2/HO-1 pathway and regulation of metabolic enzymes [78]. Similar regulatory effects on redox homeostasis have been reported for other algal polysaccharides, highlighting their potential biological relevance.

Importantly, the antioxidant activity of these compounds is strongly influenced by structural parameters such as molecular weight and degree of sulfation. Lower molecular weight polysaccharides and highly sulfated fractions have been shown to exhibit enhanced free radical scavenging activity compared to their high molecular weight or non-sulfated counterparts [79]. These observations are consistent with studies on *Ulva prolifera* and *Caulerpa lentillifera*, which confirmed that structural modification significantly impacts antioxidant efficiency [80]. In addition, ulvan extracted from *Ulva rigida* demonstrated very high DPPH scavenging activity (92.64% at 4 mg/mL), further supporting the strong antioxidant potential of green algal polysaccharides [69].

Overall, PSCT from *C. tomentosum* shows antioxidant activity that, together with its antiviral and antimicrobial properties, highlights its potential as a multifunctional natural bioactive compound.

## 3. Materials and Methods

### 3.1. Harvest Site and Preparation of Macroalgal Samples

**Figure 5:**
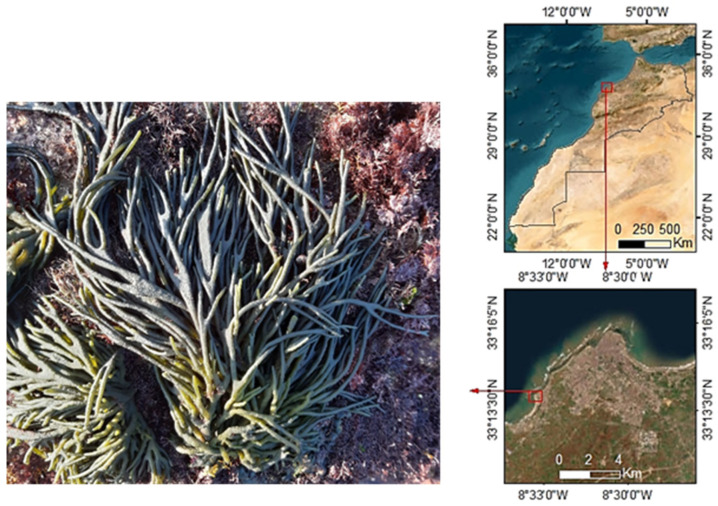
Sampling site and morphology of *C. tomentosum* (photograph by Bouchra Benhniya).

*C. tomentosum* Stackhouse, 1797 was harvested during March and April 2019 (spring) from the Atlantic coast of Sidi Bouzid, El Jadida, Morocco (33°13′55.8″ N 8°33′24.8″ W) (Figure 5). Previous studies have reported seasonal fluctuations in the biological activity of algae, with samples collected during spring showing higher bioactivity than those collected in other seasons, likely due to variations in environmental physicochemical parameters [81,82]. Sampling was performed manually during low tide at a site selected for its accessibility and the natural abundance of the species. The collected alga belongs to the class Chlorophyceae and was taxonomically identified using standard identification keys for Atlantic macroalgae, including the Determination Key for Common Algae of the Atlantic Coast and the Species Identification Guide [3]. Following collection, the samples were placed in sterile plastic bags and transported to the laboratory under refrigerated conditions (4 °C). The macroalgae were thoroughly rinsed with distilled water to remove debris and salts, then freeze-dried using a CHRIST Alpha 1-2 LD PLUS lyophilizer (Martin Christ GmbH, Osterode am Harz, Germany). The dried material was subsequently ground into a fine powder and stored under appropriate conditions until further analysis.

### 3.2. Extraction of Sulfated Polysaccharides from C. tomentosum

Sulfated polysaccharides were obtained from lyophilized *C. tomentosum* using a previously reported method with minor modifications [18]. The algal powder was first subjected to sequential treatment with organic solvents to remove pigments and lipidic compounds, followed by hot-water extraction. After concentration, the polysaccharides were precipitated with ethanol, re-dissolved in distilled water, and purified through dialysis against ultrapure water. The dialyzed extract was subsequently freeze-dried to yield crude sulfated polysaccharides (PSCT), which were stored at 20 °C until further analyses.

### 3.3. Chemical Characterization of PSCT

The extracted polysaccharide fraction (PSCT) was tested for chemical composition by running a variety of analytical assays in three replications. The total carbohydrate content was estimated using the phenol-sulfuric acid method developed by Dubois et al. [83]. The sulfate group content was estimated using the turbidimetric BaCl_2_ gelatin method as described by Dodgson and Price [18] and uronic acid concentration was estimated using the procedures established by Blumenkrantz and Asboe-Hansen [84]. Protein was measured using the Bradford method [85] and the ash content was measured by burning the samples containing polysaccharides at 600 °C for 6 h using a muffle furnace [86].

### 3.4. Characterization of PSCT

The PSCT was characterized via FTIR, UV-Vis, XRD, and SEM/EDS to assess its physicochemical properties. FTIR analysis was performed on a Thermo Scientific Nicolet iS50 spectrometer (Thermo Fisher Scientific, Waltham, MA, USA) (4000–500 cm^−1^) to identify functional groups. UV-Vis spectra were recorded from 200 to 800 nm using a PerkinElmer LAMBDA 25 spectrophotometer (PerkinElmer, Waltham, MA, USA) to detect chromophoric compounds. XRD patterns of the PSCT extract were obtained on a Bruker AXS D8 diffractometer (Bruker AXS GmbH, Karlsruhe, Germany) with Cu-Kα radiation (λ = 1.5406 Å) over 5–80° (2θ) at 2°/min to evaluate crystallinity. Morphology and elemental composition were examined via SEM-EDS (JEOL JSM-IT200 (JEOL, Tokyo, Japan) with Bruker XFlash 6/30 detector (Bruker Nano, Berlin, Germany)). Samples were gold-coated and analyzed under high vacuum at 15 kV, with elemental data expressed in weight percent (wt%). Also, the PSCT was subjected to comprehensive physicochemical characterization to elucidate its molecular and elemental composition. An HPLC system (TRACE 1300 TSQ 8000 Evo (Thermo Scientific, Waltham, MA, USA)) with a triple quadrupole mass spectrometer in positive ionization mode was used to evaluate the monosaccharide composition of the PSCT extract.

### 3.5. Biological Activities

#### 3.5.1. Antiviral Activity

##### Cell Cultures

Vero cells, which come from the kidneys of African green monkeys (ATCCCCL-81), were grown in Eagle’s Minimum Essential Medium (EMEM) containing 8% Fetal Calf Serum (FCS) and an antibiotic(s) solution (including 10,000 U penicillin, 25,000 U colimycin and 10 mg streptomycin). Cultures were observed on a regular basis to assess growth and subcultured approximately every three days.

##### Herpes Simplex Virus Stocks

Vero cell monolayers, which are flat layers of Vero cells, were infected by placing them in a 5% humidified CO_2_ atmosphere and incubating them for 2–3 days at 37 °C. After freezing and thawing the infected cells twice to lyse the cells, the cellular debris was pelleted via low-speed centrifugation and the clarified supernatant containing the virus was aliquoted and stored at −70 °C. The viral titers were determined in 96-well microtiter plates with a dilution assay expressed as 50% infectious doses (ID_50_/mL) from the cytopathic effects seen. HSV1 (wild-type strain 17, sensitive to acyclovir) was obtained from Pr. David Boutolleau (Virology Department, Hospital Pitié-Salpétrière, France).

##### Cytotoxicity and Antiviral Activity Based on Cell Viability

The cytotoxic and antiviral effects of PSCT were assessed using the neutral red uptake assay. Polysaccharide samples were prepared in MEM supplemented with 8% fetal calf serum and added to 96-well plates at a range of concentrations (1–500 μg/mL) in quadruplicate. Cells were then added (3.5 × 10^5^ cells/mL) to each well. Cells were infected (Multiplicity of Infection [MOI] = 0.001) with HSV1 in a 5% carbon dioxide (mixed with water) humidified atmosphere at 37 °C for 72 h without changing out the media in between the initial and final incubation times of the cells. The researchers included appropriate control groups for uninfected and infected cells; after completing the incubation period, the neutral red dye was assessed in each well (there was a 45 min incubation time after the addition of neutral red dye, during which time there was a significant amount of neutral red dye remaining in the wells). The incorporated dye, reflecting cell viability, was eluted with citrate buffer, and absorbance was measured at 540 nm. The data were used to determine the cytopathic effect. The 50 percent cytotoxic concentration (CC_50_) was defined as the amount of polysaccharide that decreased cell viability by 50%, while the 50 percent effective concentration (EC_50_) was defined as the amount of polysaccharide that prevented 50 percent of tissue damage caused by the virus [18,87].

#### 3.5.2. Antioxidant Activity

##### DPPH Radical Scavenging Assay

The free radical scavenging activity of PSCT was evaluated using the DPPH assay, following the method of Brand-Williams et al. [88] with slight modifications. Briefly, different concentrations of the sample (0.1–2 mg/mL) were prepared in methanol and mixed with a freshly prepared DPPH solution. The mixture was incubated in the dark for 30 min at room temperature. Absorbance was then recorded at 517 nm using a UV–Vis spectrophotometer. Ascorbic acid was used as a positive control. The radical scavenging activity was calculated as follows:

$$Scavenging\;activity\left(\%\right)=\frac{\left(Ac-As\right)}{Ac}\times 100$$

where Ac represents the absorbance of the control and As corresponds to the absorbance of the sample.

##### Ferric Reducing Antioxidant Power (FRAP) Assay

To evaluate the reducing power of PSCT, we used the methodology of Maray et al. [89], with minor modifications. In brief, 0.5 mL sample solution (0.1–2 mg/mL) was combined with 1.5 mL of phosphate buffer (0.2 M, pH 6.6) and potassium ferricyanide (1%). The resulting reaction mixture was incubated for 20 min at 50 °C, at which time trichloroacetic acid (10%) was added. After centrifugation or being allowed to settle, the amount of supernatant was 1.25 mL. This was mixed with 1.25 mL distilled water and 0.25 mL of FeCl_3_ (0.1%), incubated for 10 min at room temperature, and absorbance was determined at 700 nm. Greater absorbance means greater reducing power.

#### 3.5.3. Antimicrobial Activity

##### Inhibition of the Mycelial Growth

The effect of the PSCT on microbial growth was evaluated via kinetic monitoring of growth in liquid medium. Bacterial strains, *S. aureus* (ATCC 25923) and *E. coli* (ATCC 10536), as well as the fungal strain *C. albicans* (ATCC 60193), were cultured in Mueller–Hinton or Sabouraud broth with and without PSCT. Bacterial cultures were incubated at 37 °C for 24 h, while *C. albicans* was incubated at 30 °C for the same duration. The monitoring of microbial growth occurred through the determination of optical density at a wavelength of 600 nm using spectrophotometry. Growth curves were obtained by plotting OD600 against time, allowing the inhibitory effect of the extracts on microbial growth kinetics to be evaluated [90]. All experiments were performed under controlled conditions and in three independent replicates to ensure the reproducibility of the results.

##### Determination of the Minimum Inhibitory Concentration (MIC)

The minimum inhibitory concentration (MIC) of the PSCT tested was determined using the liquid microdilution method in 96-well plates, in accordance with standard recommendations. The microbial strains were cultured for 18–24 h, then the inoculum was adjusted to a turbidity equivalent to 0.5 McFarland and diluted to obtain a final concentration of approximately 10^5^–10^6^ CFU/mL in each well. The samples to be tested were dissolved in an appropriate solvent and then subjected to successive dilutions. Each well contained the culture medium, the microbial inoculum, and the corresponding concentration of the sample. Growth, sterility, and solvent controls were included, as well as a positive control using a reference antimicrobial agent. The plates were incubated at 30–37 °C for 18–24 h. The MIC was defined as the lowest concentration of the sample that completely inhibited visible growth of the microorganism. All experiments were performed in triplicate [36,91].

#### 3.5.4. Statistical Analysis

Mean ± Standard Deviation (SD) data are presented from two separate experimental sets of subjects. Statistical assessment was conducted with a two-factor ANOVA (Two-way analysis of variance) and Tukey’s post hoc test. An Alpha value of 0.05 (*p* < 0.05) was used to define statistical significance. GraphPad PRISM v8.4 was the software used for all statistical analyses.

## 4. Conclusions

In this study, sulfated polysaccharides extracted from the green seaweed *C. tomentosum* were successfully characterized and evaluated for their biological activities. The obtained fraction (PSCT) exhibited a complex heteropolysaccharide structure enriched in neutral sugars, sulfate groups, and uronic acids, which are known to play a key role in biological functionality. Structural analyses suggested predominantly amorphous characteristics together with the presence of functional groups typically associated with sulfated polysaccharides. Biological investigations demonstrated that PSCT possesses significant antiviral activity against HSV1, combined with strong antimicrobial effects against both bacterial and fungal pathogens. Notably, the extract exhibited high efficacy at low concentrations, particularly against *C. albicans* and *S. aureus*. In addition, PSCT showed appreciable antioxidant activity, as evidenced by its radical scavenging capacity and reducing power, suggesting its ability to mitigate oxidative stress. The multifunctional bioactivity observed in PSCT is likely attributed to its structural features, including sulfate content, monosaccharide composition, and molecular organization. These findings position *C. tomentosum* as a promising natural source of bioactive polysaccharides with potential applications in the development of novel therapeutic agents targeting viral, microbial, and oxidative stress-related disorders. However, further studies are required to isolate specific active fractions, determine their molecular mechanisms of action, and evaluate their efficacy in vivo models. Overall, this work provides new insights into the valorization of green seaweed polysaccharides and supports their potential use in pharmaceutical and biomedical applications.

## Figures and Tables

**Table 1:** Chemical composition and monosaccharide composition of PSCT.

Content (%. *w*/*w*) of PSCT	
Yields	22.01 ± 0.03
Ash	3.07 ± 0.11
Total neutral sugars	66.84 ± 0.03
Sulfates groups	8.73 ± 0.16
Uronic acids	4.13 ± 0.05
Proteins content	1.14 ± 0.10
**Monosaccharide (% of total sugars)**	
Galactose	51.88 ± 0.13
Arabinose	32.64 ± 2.08
Xylose	4.28 ± 0.22
Rhamnose	2.31 ± 0.64
Glucuronic acid	6.62 ± 1.18
Fructose	0.93 ± 0.03

**Table 2:** Cytotoxicity and antiviral effects (EC_50_) of PSCT fraction.

	CC_50_ (μg/mL)	EC_50_ (μg/mL)
**PSCT**	>200.00	12.22 ± 2.9
**Acyclovir**	>200.00	0.58 ± 0.23

**Table 3:** Minimum inhibitory concentration (MICs) of PSCT from *C. tomentosum*.

Microorganism	MIC (mg/mL)
*S. aureus* (ATCC 25923)	0.10 ± 0.01
*E. coli* (ATCC 10536)	0.78 ± 0.2
*C. albicans* (ATCC 60193)	0.05 ± 0.01

## Data Availability

The original contributions presented in the study are included in the article. Further inquiries can be directed to the corresponding author.
